# Lipidomics Reveals Myocardial Lipid Composition in a Murine Model of Insulin Resistance Induced by a High-Fat Diet

**DOI:** 10.3390/ijms25052702

**Published:** 2024-02-26

**Authors:** Josefa Girona, Oria Soler, Sara Samino, Alexandra Junza, Neus Martínez-Micaelo, María García-Altares, Pere Ràfols, Yaiza Esteban, Oscar Yanes, Xavier Correig, Lluís Masana, Ricardo Rodríguez-Calvo

**Affiliations:** 1 Vascular Medicine and Metabolism Unit, Research Unit on Lipids and Atherosclerosis, “Sant Joan” University Hospital, Institut de Investigació Sanitaria Pere Virgili (IISPV), Universitat Rovira i Virgili, 43204 Reus, Spain; 2 Spanish Biomedical Research Centre in Diabetes and Associated Metabolic Disorders (CIBERDEM), Institute of Health Carlos III, 28029 Madrid, Spain; 3 Metabolomics Platform, Department of Electronic Engineering (DEEEA), Universitat Rovira i Virgili, 43002 Tarragona, Spain

**Keywords:** myocardial steatosis, lipid peroxidation, cardiac lipotoxicity

## Abstract

Ectopic fat accumulation in non-adipose tissues is closely related to diabetes-related myocardial dysfunction. Nevertheless, the complete picture of the lipid metabolites involved in the metabolic-related myocardial alterations is not fully characterized. The aim of this study was to characterize the specific lipid profile in hearts in an animal model of obesity/insulin resistance induced by a high-fat diet (HFD). The cardiac lipidome profiles were assessed via liquid chromatography–mass spectrometry (LC–MS)/MS-MS and laser desorption/ionization–mass spectrometry (LDI–MS) tissue imaging in hearts from C57BL/6J mice fed with an HFD or standard-diet (STD) for 12 weeks. Targeted lipidome analysis identified a total of 63 lipids (i.e., 48 triacylglycerols (TG), 5 diacylglycerols (DG), 1 sphingomyelin (SM), 3 phosphatidylcholines (PC), 1 DihydroPC, and 5 carnitines) modified in hearts from HFD-fed mice compared to animals fed with STD. Whereas most of the TG were up-regulated in hearts from animals fed with an HFD, most of the carnitines were down-regulated, thereby suggesting a reduction in the mitochondrial β-oxidation. Roughly 30% of the identified metabolites were oxidated, pointing to an increase in lipid peroxidation. Cardiac lipidome was associated with a specific biochemical profile and a specific liver TG pattern. Overall, our study reveals a specific cardiac lipid fingerprint associated with metabolic alterations induced by HFD.

## 1. Introduction

Ectopic fat accumulation in non-adipose tissues is closely related to an altered cardiac structure, impaired contractile function, both systolic and diastolic myocardial dysfunction, and increased risk for cardiometabolic events [1,2,3,4,5]. Actually, it has been reported that myocardial lipid accumulation precedes myocardial dysfunction [3,5]. Thereby, ectopic fat depots may be clinically useful to identify individuals at increased cardiometabolic risk [6]. Functional alterations of epicardial adipose tissue found in obese and diabetic patients have been linked to both vascular and myocardial dysfunction [7,8]. Additionally, the intramyocellular build-up of inert triacylglycerol stores and bioactive lipid metabolites in cardiac cells regulates cellular signaling pathways leading to an altered cardiac function related to insulin resistance [9,10]. Apart from myocardial lipid content, hepatic steatosis has been found independently associated with myocardial dysfunction [11]. In fact, cardiovascular (CV) disease is the leading cause of mortality in patients with non-alcoholic fatty liver disease (NAFLD) [12,13], and patients with NAFLD are at two-fold risk of dying of CV disease than liver disease [14]. Therefore, the cardiometabolic risk related to fat content is the result not only of the ectopic fat levels but also of its localization.

Clinical determination of the fat content has been performed via proton magnetic resonance spectroscopy (1H-MRS) during last decade [15]. However, this approach is cost-expensive and requires specialized centers and personnel, so it is not suitable for a large screening population. Furthermore, it does not allow us to discern between the type and distribution of myocardial fat, so the fat composition of the myocardium of individuals with metabolic disorders is not fully characterized. Since certain bioactive lipid metabolites, such as triacylglycerols (TG), diacylglycerols (DG), or ceramides (Cer), among others, can activate cellular signaling pathways, leading to the activation of an altered metabolic response [16,17,18,19], the myocardial fat characterization in metabolic individuals is of special relevance.

Here, we analyze the myocardial fat composition in a murine model of obesity/insulin resistance induced by a high-fat diet (HFD) using both liquid chromatography–mass spectrometry (LC–MS) and laser desorption/ionization–mass spectrometry (LDI–MS) tissue imaging approaches. Furthermore, we evaluate the potential associations of the most representative cardiac metabolites with both the biochemical plasma variables and liver TG.

## 2. Results

### 2.1. HFD Induces Ectopic Fat Accumulation in Heart

**Figure 1:**
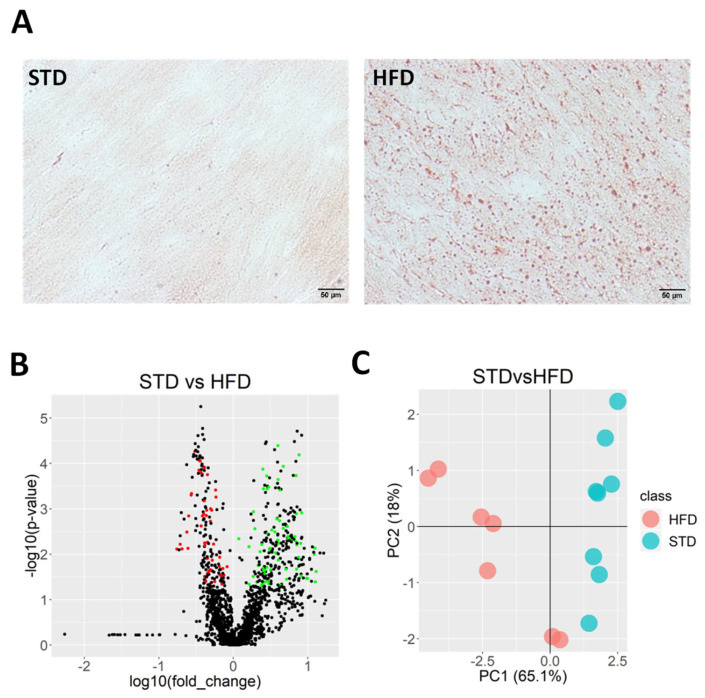
HFD induced myocardial lipid accumulation in C57BL/6J mice. (**A**) Representative Oil Red O staining in hearts cross sections from mice fed with STD or HFD. Scale bar: 50 μm. (**B**) Volcano plot of all hits in hearts from STD- and HFD-fed animals. Colored points indicate metabolites that were significantly down- (red) or upregulated (green) in hearts from HFD-fed animals compared to hearts from animals fed with STD. (**C**) PCA score plots from hearts of STD- and HFD-fed animals.

The HFD increased body weight (28%, *p* < 0.001) and induced both fasting blood glucose (~2.1-fold, *p* < 0.001) and the homeostatic model assessment (HOMA) insulin resistance index (~3.9-fold, *p* < 0.05) compared with control mice fed with a standard diet (STD) [20,21,22,23]. Oil Red O staining clearly showed a greater lipid deposition in the hearts of animals fed with an HFD than in the hearts of STD-fed mice (Figure 1A). In order to explore the myocardial lipid composition in our model, an untargeted approach using LC/MS analyses identified a total of 188 metabolites differentially expressed between the hearts from STD- and HFD-fed animals (Figure 1B). To obtain a preliminary picture of the overall differences in the cardiac metabolites between both study groups, an exploratory PCA, including the differentially expressed hits, was performed. The first (PC1)—but not the second (PC2)—component clearly classified the STD- and HFD-fed animals, explaining 61.5% of the variance in the data (Figure 1C).

### 2.2. Lipidomics Reveals Myocardial Lipid Composition in HFD-Fed Mice

**Figure 2:**
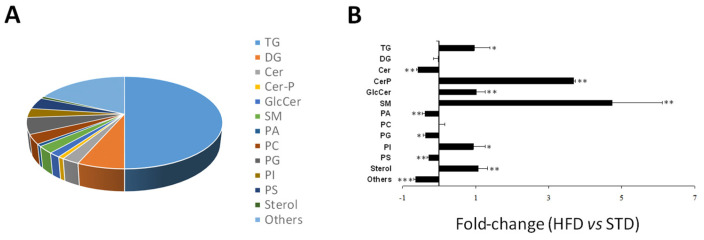
Family lipid profile in hearts from HFD-fed mice. (**A**) Pie chart of the lipid class identified in hearts from HFD-mice. (**B**) Changes in total signal of the different lipid species in hearts from HFD-fed mice compared with mice fed with a STD. TG: triacylglycerols; DG: diacylglycerols; Cer: ceramides; Cer-P ceramide phosphate; GlcCer; glucoceramides; SM: sphingomyelins; PA: phosphatidic acid; PC: phosphatidylcholines; PG: phosphatidylglycerols; PI: phosphatidylinositols; PS: phosphatidylserines. Data are expressed as the mean ± SEM (* *p* < 0.05, ** *p* < 0.01, *** *p* < 0.001 vs. STD-fed mice).

Putative identification of the metabolites differentially found between both groups classified 66 of them in more than one lipid family, so they were excluded in the following analyses. Of the 122 metabolites classified in a single family, 61 (~50.00%) were TG and 8 (6.56%) were DG. An amount of 4.92% of total Cer were identified (including three ceramides, one ceramide phosphate (Cer-P), and two glucoceramides (GlcCer)), as well as a 2.46% of sphingomyelins (SM). A total of 17.21% were identified as phospholipids (including one phosphatidic acid (PA), four phosphatidylcholines (PC), seven phosphatidylglycerols (PG), four phosphatidylinositols (PI), and five phosphatidylserines (PS)) and 0.82% as sterols. Finally, 22 compounds (18.03%) were classified as others lipid species (Figure 2A). Taken together, the total amount of TG was up-regulated in the heart from HFD-fed animals compared to animals fed with STD (Figure 2B). However, whereas most of the TG were up-regulated, nine of them were found down-regulated in the hearts of animals fed with HFD. No changes were found in the total amount of myocardial DG (Figure 2B), but half of them were up-regulated and the other half were down-regulated in the hearts of the HFD-fed animals, whereas Cer were down-regulated in the HFD-fed animals, both Cer-P and GlcCer, as well as SM, were up-regulated in the hearts from animals fed with the HFD (Figure 2B). HFD reduced myocardial PA, PG, and most PS and induced most of the PI (Figure 2B). Although most of the PC were down-regulated in hearts from the HFD-fed animals, no changes were found in the total amount of PC between both groups (Figure 2B). Myocardial sterol levels were up-regulated by the HFD, and the total amount of others lipid species were down-regulated in hearts from the HFD-fed animals (Figure 2B).

**Figure 3:**
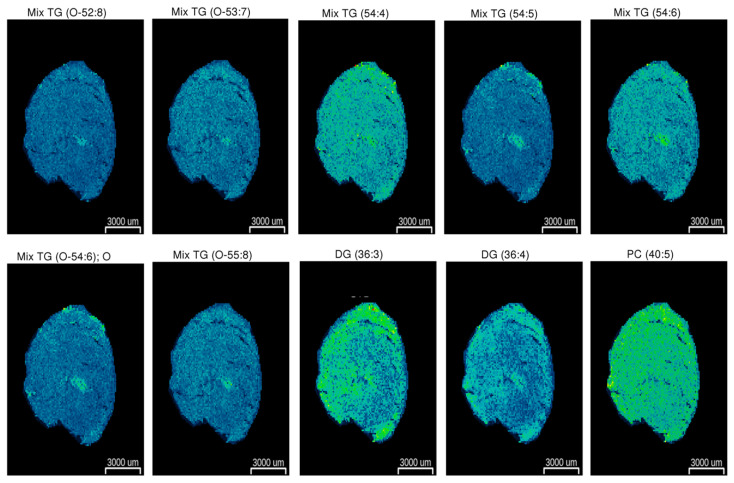
LDI–MS tissue imaging visualization of the metabolite distributions in heart cross sections. A representative tissue image showing the abundance of 10 of the 63 metabolites identified via LC-MS is shown. Green color denotes a higher abundance of a particular metabolite, whereas blue denotes a lower abundance. Average spectra identifying the Mix TG (O-52:8): *m*/*z* 855.68, mass error Δ = −39 ppm; Mix TG (O-53:7): *m*/*z* 871.72, mass error Δ = 35 ppm; Mix TG (54:4): *m*/*z* 905.76, mass error Δ = 17 ppm; Mix TG (54:5): *m*/*z* 881.76, mass error Δ = 28 ppm; Mix TG (54:6): *m*/*z* 879.74, mass error Δ = 33 ppm; Mix TG (O-54:6):O: *m*/*z* 881.76, mass error Δ = 28 ppm; Mix TG (O-55:8): *m*/*z* 897.73, mass error Δ = 42 ppm; DG (36:3): *m*/*z* 641.51, mass error Δ = 24 ppm; DG (36:4): *m*/*z* 617.51, mass error Δ = 22 ppm; PC (40:5): *m*/*z* 820.62, mass error Δ = 25 ppm.

Targeted MS/MS analysis identified a total of 63 lipids, including 48 TG, 5 DG, 1 SM, 3 PC, 1 DihydroPC, and 5 carnitines (Table 1). The myocardial lipid profile obtained by LC/MS was further validated using LDI-MS tissue imaging. Following this approach, 10 of these 63 lipids were further identified in heart cross sections via LDI–MS tissue imaging (Figure 3).

### 2.3. Identified Metabolites Classify Both the STD and the HFD Groups

**Figure 4:**
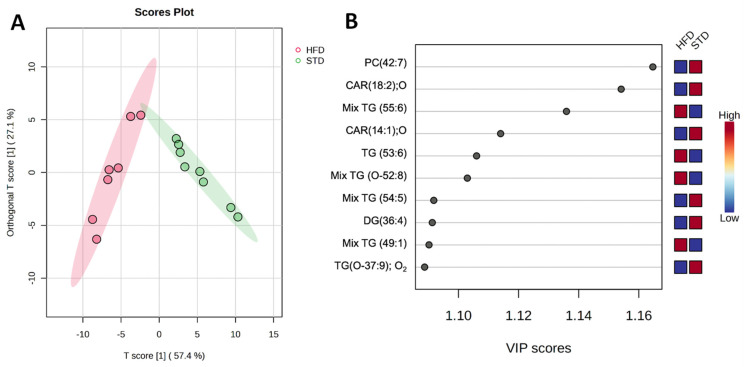
OPLS-DA score plots (**A**) and OPLS-DA-derived VIP values (**B**) performed with the identified metabolites in hearts from STD and HFD mice. The blue boxes denote lower abundance of a particular metabolite, whereas red boxes denote a higher abundance.

To explore the classification of the two groups by the identified metabolites, the OPLS-DA supervised method was used. OPLS-DA showed a clear group separation (R2Y = 0.735; Q2 = 0.694) (Figure 4A). In order to explore which of the identified metabolites better classify both groups, the VIP values of the OPLS-DA model was employed. Figure 4B showed the top-ten metabolites that better classify the two groups. Whereas PC (42:7), CAR (18:2); O, CAR (14:1); O, Mix TG (54:5), DG (36:4), and TG (O-37:9); O_2_ were enriched in the hearts of animals fed with STD, Mix TG (56:6), TG (53:6), Mix TG (O-52:8) and Mix TG (49:1) were found enriched in hearts from the HFD-fed animals (Figure 4B).

### 2.4. Myocardial Lipid Composition Correlates to Plasma Variables

**Figure 5:**
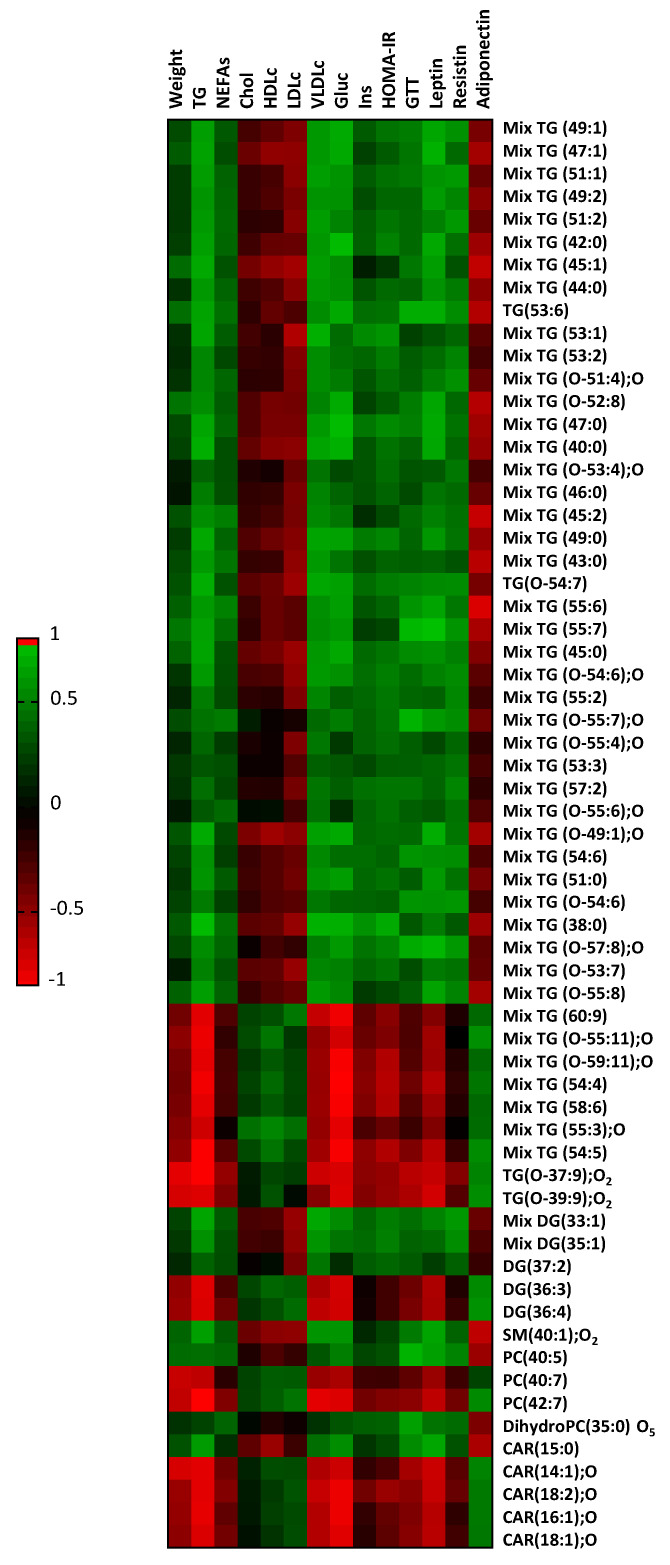
Heatmap showing Spearman’s correlation between the altered myocardial metabolites and the weight and biochemical variables related to glucose and fatty acid metabolism. The color intensity shows the degree of the association, with the positive correlations in green and the negative ones in red.

Next, the potential associations among the myocardial lipid composition and physiological parameters related to glucose and fatty acid metabolism were assessed (Figure 5, Supplementary Table S1). Most of the identified metabolites significantly correlated to selected plasma variables. Specifically, 51 of the were found correlated to plasma triglycerides, 45 with very low-density lipoprotein cholesterol (VLDLc), 44 with glucose, 41 with leptin, and 24 with adiponectin. However, whereas positive correlations were found among the abovementioned first four variables and the upregulated metabolites found in the hearts from the HFD-fed mice, they were inversely correlated with adiponectin. Conversely, the metabolites found down-regulated in hearts from animals fed with the HFD directly were correlated with adiponectin and inversely correlated with the other variables (i.e., triglycerides, VLDLc, glucose, and leptin).

### 2.5. Liver Fat Content Is Related to Myocardial Lipid Composition

**Figure 6:**
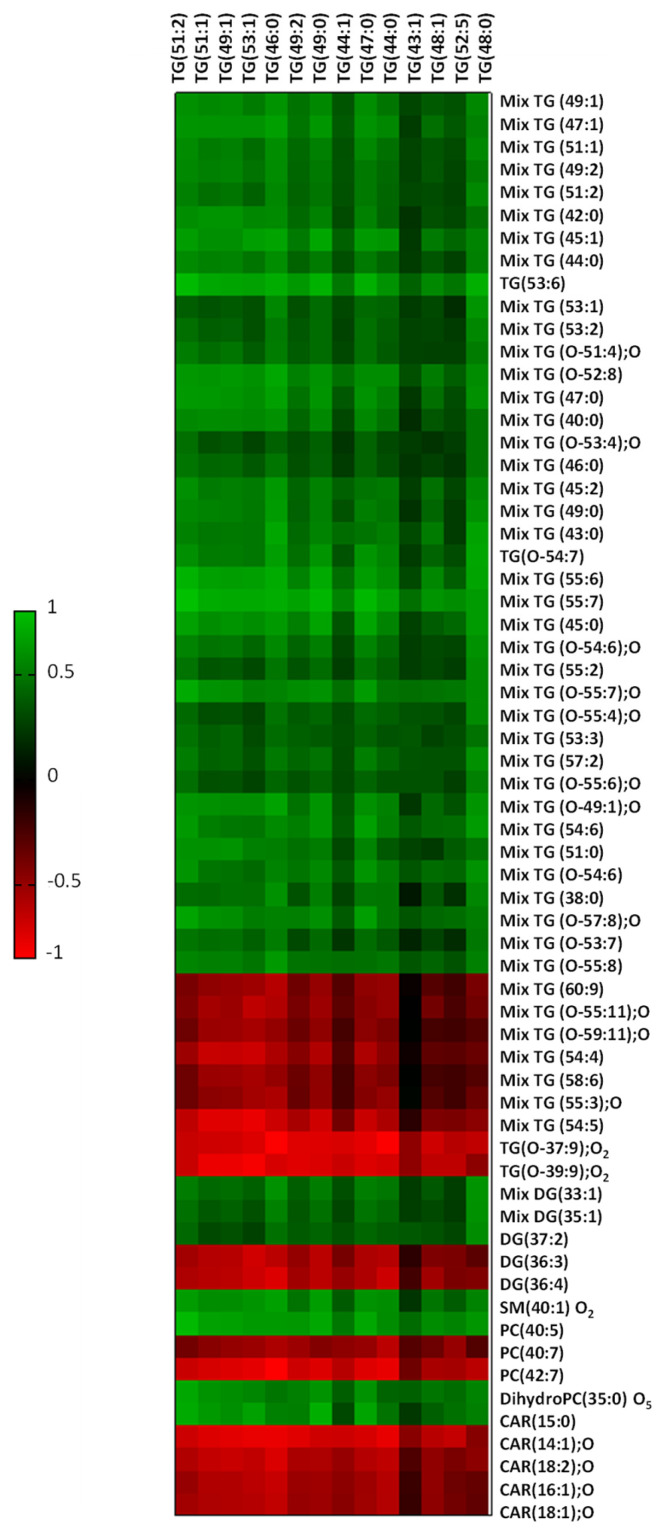
Heatmap showing Spearman’s correlation between the altered myocardial metabolites and liver TG. The color intensity shows the degree of the association, with the positive correlations in green and the negative ones in red.

Finally, we explored the potential associations among the myocardial lipid composition and the specific liver triglyceride pattern previously identify in our animal model [21] (Figure 6, Supplementary Table S2). Positive correlations were found among liver triglyceride and the HFD-upregulated cardiac metabolites. Nevertheless, cardiac metabolites downregulated by the HFD were inversely correlated to liver TG.

## 3. Discussion

Ectopic fat accumulation in non-adipose tissues is related to an increased risk for CV events [1,2,3,4,5]. Increasing evidence propose cardiac lipid accumulation as one of the main precursors for the myocardial dysfunction due to diabetes [2,3,4,5,24], thereby suggesting a lipotoxic process underlying the cardiac functional alterations of diabetic etiology. Actually, it has been reported that stores of triacylglycerol and other bioactive lipid metabolites in cardiac cells impairs both insulin-stimulated glucose uptake [9] and oxidation [10]. Nevertheless, the full picture of lipid metabolites involved in the metabolic disturbances related to myocardial dysfunction is not fully depicted. Here, we identified a myocardial lipid fingerprint related to metabolic alterations in a murine model of obesity/insulin resistance induced by an HFD [22,23].

Characterization of our model was described elsewhere [20,21,22,23]. Briefly, our HFD-induced insulin resistance model showed higher body weight, fasting blood glucose, and HOMA-insulin resistance index than the STD-fed animals. Interestingly, an increase in the myocardial lipid accumulation had been already observed in the animals fed with an HFD compared to STD-fed animals [22,23]. In order to fully characterize the myocardial lipid composition in these animals, an untargeted approach was performed. Roughly 50% of the differentially-found metabolites between both groups were identified as putative TG, highlighting the prevalent role of this lipid specie in the cardiac lipidomic remodeling induced by the HFD. Actually, the total TG species pool had been previously found to be increased in myocardium in both obese/type 2 diabetes patients [2,3,5,25,26,27,28] and murine models of diet-induced obesity/insulin resistance [29,30,31,32,33,34,35,36]. Specifically, our targeted analysis identified 39 TG up-regulated in hearts from HFD-fed mice. These data are in line with a recent study reporting that 11 kinds of TG increased in a mice model of diabetic cardiomyopathy [35]. Additionally, 9 TG were found down-regulated by the HFD, thereby suggesting that the myocardial lipid composition is subjected to complex dynamics. Although it was suggested that TG are metabolically inactive [37], myocardial TG have been related to diastolic dysfunction [5,38,39] and impaired myocardial strain in patients with type 2 diabetes [4,38]. Additionally, increasing evidence has linked TG accumulation with insulin resistance [40,41,42]. Apart from TG, we found that HFD also modified the content of DG, SM, PC, and carnitines to a lesser extent, thereby highlighting that lipid species others than TG may also have a relevant role in lipid-induced cardiac adaptation. Actually, DG accumulation in myocardium has been found in both diabetic patients [43] and animal models [33,44], and it has been related to cardiac lipotoxicity [18,45,46,47]. DG are involved in cellular signaling, including the PKCθ-induced inhibitory phosphorylation of IRS1 [48,49,50], thereby acting as second messengers leading to insulin resistance [9,51,52]. The DG have been further related to cardiac fibrosis and heart failure [36,53,54,55,56], potentially through PKC-induced oxidative stress [53,54]. DG may be produced from SM. SM are associated with lipid rafts in the cell membranes, which take part in the insulin resistance-related signal transduction [57,58]. The role of these molecules in the hearts of animal models fed with an HFD have yielded somewhat conflicting results. While SM was to be found reduced in hearts from HFD-fed rats [36], two kinds of sphingomyelin were found to have increased in hearts from streptozotocin-induced diabetic mice fed with an HFD [35]. Our data revealed one SM induced in the heart from the HFD-fed mice, thereby supporting the SM involvement in the HFD-induced metabolic disturbances related to diabetes. PC, one of the main components of the biological membranes involved in the cholesterol esterification of the high-density lipoproteins (HDL), was not modified in previous studies [36]. Our data reveal a variable response of PC to HFD, so further studies are necessary in order to clarify the role of these molecules in the insulin resistance heart. Carnitines are rate-limiting factors for energy production from long-chain fatty acids, regulating its transport to mitochondria for β-oxidation [59]. Although the diabetic heart shows an initial compensatory increase in fatty acid oxidation, the myocardial lipid accumulation suggests an impaired mitochondrial function in later stages. Therefore, the carnitines reduction found in our animal model may be related to mitochondrial impairment and the increase in the TG levels observed in the hearts from animals fed with the HFD. Taken together, the HFD-related lipidomic profile may directly impact the lipotoxic-induced myocardial dysfunction. Actually, ~30% of the identified metabolites were oxidated, with the polyunsaturated fatty acids found in the different lipid species being the most susceptible to lipid peroxidation. It is worth noting that although saturated and monounsaturated fatty acids were also found, most of the fatty acids were long-chain polyunsaturated fatty acids, potentially due to the type of diet used.

Although lipidomics has emerged as a powerful approach for the identification of specific lipid species related to the cardiac metabolic dysfunction related to diabetes, it is worth noting that the different methods for compound identification have yielded somewhat conflicting results. Therefore, a standardization of the methodological approaches among the different laboratories is necessary for the study comparison. In order to validate our data, we monitored the spatial distribution of the identified compounds via LDI/MS tissue imaging. Ten of the 63 lipids identified by MS/MS could be further detected in heart cross sections, showing a relative homogeneous distribution. Nevertheless, further research is warranted in order to identify potentially different metabolites distribution among the different areas of heart (i.e., atriums vs. ventricles).

Because the lipid profile found in the hearts of the HFD-fed animals includes metabolites involved in altered metabolic profiles, such as insulin resistance [9,51,57,58], we next explored the potential associations among the identified cardiac metabolites and variables related to glucose and fatty acid metabolism. We have previously shown that the total amount of cardiac TG directly correlated with plasma levels of glucose, triglycerides, VLDL, leptin, and resistin [23]. In line with these observations, most of the identified up-regulated metabolites (including TG) were positively correlated with the above-mentioned variables, including leptin and resistin, but inversely correlated with plasma adiponectin. Conversely, the metabolites down-regulated by the HFD were positively correlated with adiponectin and inversely correlated with the other variables (i.e., plasma levels of glucose, triglycerides, VLDL, leptin, and resistin). Therefore, our data suggest that a poor plasmatic biochemical profile may be indicative of an altered cardiac lipid profile. Actually, it is well known that the plasma biochemical profile of diabetic patients is related to the development of metabolic alterations in the different target tissues, including the heart. The non-esterified fatty acids (NEFAs), as well as the fatty acids found esterified in the liver-released triglyceride-rich lipoproteins, are associated with an increased fatty-acid uptake by the cardiac cells. This leads to the accumulation of bioactive lipid intermediates, such as those identified in the current work, resulting in the regulation of several cellular signaling pathways involved in the imbalance of cellular responses, including insulin-mediated glucose uptake, endoplasmic reticulum stress, inflammation, mitochondrial dysfunction, oxidative stress, and fibrosis or apoptosis, among others. As mentioned above, PKC activation by several of these bioactive lipid mediators has been related to some of these process [36,48,49,50,53,54,55,56]. Additionally, hyperglycaemia and adipokines, such as leptin and resistin, have also been related to the development of insulin resistance in different tissues. Therefore, an altered biochemical profile could be indicative of alterations in the myocardium related to heart failure in diabetic individuals. In fact, diabetic individuals have an increased risk of heart failure independently of hypertension, coronary artery disease, and valvular heart disease [60], although the molecular mechanisms underlying this risk are not fully known. Our data suggest that ectopic fat accumulation in the heart is related to a state of oxidative stress and lipid peroxidation. Therefore, the plasma biochemical profile could be indicative of the oxidative state in the heart of diabetic individuals, allowing the initiation of therapies aimed at reducing these processes, for example, with the use of antioxidants. Additionally, given the role of PKC in the lipid-induced cellular responses, therapies aimed at inhibiting this kinase may be also considered. Since the accumulation of ectopic fat in the heart precedes the development of myocardial dysfunction [3,6], these types of therapies could contribute to slowing the progression of the disease.

Given that liver steatosis is closely related to an altered cardiac function [11], we finally explored the potential associations among the myocardial lipid composition and the specific liver TG pattern previously identified in our animal model [21]. Similar to data from the biochemical plasma profile, whereas most of the cardiac metabolites up-regulated by the HFD were directly associated with most of the liver TG, these latest were inversely correlated with the HFD-downregulated cardiac metabolites. Thus, the HFD-induced altered lipidomic profiles found in heart and liver may be somewhat related. Actually, the liver is the main source of triglyceride-rich lipoproteins, which are released to bloodstream, acting as interlocutors between the liver and the peripheral tissues, including the heart. Given that the hepatic de novo lipogenesis exceeds the VLDL production in metabolic individuals [61,62], the liver TG profile may influence the myocardial lipid composition through the liver-derived triglyceride-rich lipoproteins. Nevertheless, further research is warranted in order to determine whether metabolic disturbances found in one of the tissues may influence the other one.

## 4. Materials and Methods

### 4.1. Animal Model Experiments

Six-week-old C57BL/6J mice were randomly distributed inro two experimental groups and fed ad libitum a standard chow diet (STD: 10% kcal from fat; Panlab; Barcelona, Spain) or a high-fat diet (HFD: 60% kcal from fat; Panlab; Barcelona, Spain) for 12 weeks in standard light–dark cycle (12 h light/dark cycle) and temperature (21 ± 1 °C) conditions [20,21,22,23]. No changes were found in the food intake between the experimental groups. Animals were euthanized, and the heart and the liver were removed, frozen in liquid nitrogen, and stored at −80 °C. Experiments were conformed according to the Guide for the Care and Use of Laboratory Animals published by the U.S. National Institutes of Health (NIH publication no. 85-23, revised 1996). All procedures were approved by the University Rovira i Virgili Bioethics Committee, as stated in Law 5/21 July 1995 passed by the Generalitat de Catalunya (Autonomous Government of Catalonia).

### 4.2. Biochemical Plasma Profile

Animals were fasted for 4h, and a blood sample was collected. Plasma samples were analyzed for triglycerides, total cholesterol, HDL cholesterol (HDLc), glucose (Spinreact; Barcelona, Spain), and non-esterified fatty acids (NEFAs) (Wako; Osaka, Japan) via standardized colorimetric methods adapted to the Cobas Mira Plus Autoanalyzer (Roche Diagnostics, Barcelona, Spain). The low-density lipoprotein cholesterol (LDLc) concentration was calculated using the Friedewäld formula, and the concentration of VLDLc was determined by the formula total cholesterol—(HDLc + LDLc). Plasma levels of insulin, resistin, leptin, and adiponectin were determined by commercial ELISA Kits (Milliplex^®^, Millipore; Billerica, MA, USA). The homeostatic model assessment (HOMA) index was calculated as previously described [23,63].

### 4.3. Lipid Staining

Lipid staining was performed in 5 µm cross-sections from frozen mouse hearts using Oil Red O staining following the protocol described by Mehlem et al. [64]. Pictures were captured using a microscope (Olympus IX71, Barcelona, Spain).

### 4.4. Lipidomics

Lipid extraction and fragmentation was performed as previously described [21] and the organic phase was collected, dried under a stream of nitrogen, and resuspended in methanol–toluene (9:1) for LC/MS analysis.

Untargeted LC/MS analyses were performed as previously described [21] using a UHPLC system (1200 series, Agilent Technologies, Santa Clara, CA, USA) coupled to a 6550 ESI-QTOF MS (Agilent Technologies) operating in positive (ESI+) electrospray ionization mode. For compound identification, MS/MS analyses were performed in targeted mode (*m*/*z* range from 700–900, with a default iso width of 4 *m*/*z* and collision energy at 20 V). Lipid structures were identified by matching tandem MS spectra against reference standards in LIPID MAPS [65] and/or LipidBlast [66] and/or Metlin (https://metlin.scripps.edu (accessed on 3 February 2022)) databases. Data were processed using XCMS software (version 1.34.0) [67] and normalized by dry weight.

### 4.5. Tissue Molecular Imaging

Tissue imaging via laser desorption/ionization–mass spectrometry (LDI–MS) imaging was performed as previously described [21,68,69] in 10 µm heart sections covered by gold monolayers. LDI–MS tissue images were acquired using a MALDI-TOF UltrafleXtreme instrument with SmartBeam II Nd:YAG/355 nm laser (Bruker Daltonics, Billerica, MA, USA) and processed by using open-source software rMSI (version 0.3.1) [70] and rMSIproc (https://github.com/prafols/rMSIproc (version 0.9.1) (accessed on 2 July 2022)) [69]. The metabolites’ tentative identification was based on the exact mass of their sodium adduct according to the Human Metabolome Database, filtering metabolites with mass error Δ < 50 ppm, and the results obtained from the LC–MS/MS experiments.

### 4.6. Statistical Analysis

Data are expressed as the mean ± standard error of the mean (SEM), and significant differences between groups were established using Student’s *t*-test. Volcano plots were obtained using a fold change threshold of 2 and a p value threshold of 0.05. Principal components analysis (PCA) was performed to describe the metabolic profiles between groups. The loading analysis of the principal components was used to identify the most relevant molecular components. The orthogonal partial least squares discriminant analysis (OPLS-DA) supervised regression modeling was applied for group discrimination. The OPLS-DA validity and predictive ability were estimated via the R2Y parameters and Q2 values, respectively. The variable importance in the projection (VIP) values of the OPLS-DA model was used to identify which metabolites better classify the groups. A correlation between differential metabolites and physiological parameters was performed via Spearman’s test. Statistical analyses were performed using SPSS software (IBM SPSS Statistics, version 22.0). Differences were considered statistically significant at *p* < 0.05.

## 5. Conclusions

Altogether, our findings show that HFD induces changes in the cardiac lipidomic profile, mainly characterized by an increase in the number of TG and a decrease in several carnitines, among other molecules. This cardiac metabolic profile may indicate the reduction in the fatty acids mitochondrial β-oxidation as one of the potential underlying mechanisms involved in the ectopic fat accumulation in the myocardium. In addition, the increase in lipid peroxidation suggests the onset of a lipotoxic process related to the worsening cardiac function in individuals with metabolic disorders. Finally, the cardiac lipidome paralleled the alterations in the plasma biochemical profile and a specific liver TG pattern, thereby suggesting that the myocardial lipid disturbances are closely related to other metabolic disturbances, both at the systemic level and at the level of specific tissues.

## Figures and Tables

**Table 1:** Heart metabolites upregulated in HFD-fed mice. The accuracy for the *m*/*z* values reported is <0.005 ppm.

					HFD vs. STD	
	*m*/*z*	Metabolites	Ion	Formula	Fold	*p*-Value
TG						
	836.7700	Mix TG (49:1)	NH_4_^+^	C_52_H_98_O_6_	16.37	0.026
	808.7379	Mix TG (47:1)	NH_4_^+^	C_50_H_94_O_6_	12.94	0.030
	864.8008	Mix TG (51:1)	NH_4_^+^	C_54_H_102_O_6_	12.45	0.023
	834.7538	Mix TG (49:2)	NH_4_^+^	C_52_H_96_O_6_	12.37	0.032
	862.7855	Mix TG (51:2)	NH_4_^+^	C_54_H_100_O_6_	12.21	0.021
	740.6752	Mix TG (42:0)	NH_4_^+^	C_45_H_86_O_6_	9.94	0.042
	780.7065	Mix TG (45:1)	NH_4_^+^	C_48_H_90_O_6_	9.61	0.029
	768.7070	Mix TG (44:0)	NH_4_^+^	C_47_H_90_O_6_	8.15	0.037
	887.7088	TG (53:6)	Na^+^	C_56_H_96_O_6_	7.63	0.003
	892.8326	Mix TG (53:1)	NH_4_^+^	C_56_H_106_O_6_	7.53	0.026
	890.8169	Mix TG (53:2)	NH_4_^+^	C_56_H_104_O_6_	7.13	0.018
	860.7692	Mix TG (O-51:4); O	NH_4_^+^	C_54_H_98_O_6_	7.08	0.024
	855.6826	Mix TG (O-52:8)	Na^+^	C_55_H_92_O_5_	5.42	0.006
	810.7533	Mix TG (47:0)	NH_4_^+^	C_50_H_96_O_6_	5.33	0.014
	712.6440	Mix TG (40:0)	NH_4_^+^	C_43_H_82_O_6_	5.18	0.036
	888.8010	Mix TG (O-53:4); O	NH_4_^+^	C_56_H_102_O_6_	5.09	0.026
	796.7386	Mix TG (46:0)	NH_4_^+^	C_49_H_94_O_6_	4.36	0.041
	778.6904	Mix TG (45:2)	NH_4_^+^	C_48_H_88_O_6_	4.22	0.030
	838.7852	Mix TG (49:0)	NH_4_^+^	C_52_H_100_O_6_	4.03	0.018
	754.6908	Mix TG (43:0)	NH_4_^+^	C_46_H_88_O_6_	4.02	0.029
	885.7314	TG(O-54:7)	Na^+^	C_57_H_98_O_5_	3.98	0.005
	915.7406	Mix TG (55:6)	Na^+^	C_58_H_100_O_6_	3.96	0.001
	913.7245	Mix TG (55:7)	Na^+^	C_58_H_98_O_6_	3.83	0.004
	782.7220	Mix TG (45:0)	NH_4_^+^	C_48_H_92_O_6_	3.69	0.020
	898.7885	Mix TG (O-54:6); O	NH_4_^+^	C_57_H_100_O_6_	3.29	0.015
	923.8039	Mix TG (55:2)	Na^+^	C_58_H_108_O_6_	3.13	0.014
	910.7853	Mix TG (O-55:7); O	NH_4_^+^	C_58_H_100_O_6_	3.04	0.016
	916.8320	Mix TG (O-55:4); O	NH_4_^+^	C_58_H_106_O_6_	2.99	0.036
	893.7564	Mix TG (53:3)	Na^+^	C_56_H_102_O_6_	2.81	0.018
	951.8339	Mix TG (57:2)	Na^+^	C_60_H_112_O_6_	2.64	0.019
	912.7994	Mix TG (O-55:6); O	NH_4_^+^	C_58_H_102_O_6_	2.54	0.032
	843.7401	Mix TG (O-49:1); O	Na^+^	C_52_H_100_O_6_	2.53	0.007
	901.7247	Mix TG (54:6)	Na^+^	C_57_H_98_O_6_	2.50	0.016
	866.8155	Mix TG (51:0)	NH_4_^+^	C_54_H_104_O_6_	2.41	0.013
	887.7463	Mix TG (O-54:6)	Na^+^	C_57_H_100_O_5_	2.41	0.036
	689.5683	Mix TG (38:0)	Na^+^	C_41_H_78_O_6_	2.39	0.029
	936.8010	Mix TG (O-57:8); O	NH_4_^+^	C_60_H_102_O_6_	2.28	0.005
	871.7145	Mix TG (O-53:7)	Na^+^	C_56_H_96_O_5_	1.62	0.006
	897.7299	Mix TG (O-55:8)	Na^+^	C_58_H_98_O_5_	1.19	0.009
	979.7707	Mix TG (60:9)	Na^+^	C_63_H_104_O_6_	0.56	0.007
	902.7280	Mix TG (O-55:11); O	NH_4_^+^	C_58_H_92_O_6_	0.52	0.007
	958.7905	Mix TG (O-59:11); O	NH_4_^+^	C_62_H_100_O_6_	0.49	0.009
	905.7571	Mix TG (54:4)	Na^+^	C_57_H_102_O_6_	0.49	0.001
	957.7873	Mix TG (58:6)	Na^+^	C_61_H_106_O_6_	0.47	0.009
	953.7562	Mix TG (55:3); O	K^+^	C_58_H_106_O_7_	0.47	0.005
	903.7412	Mix TG (54:5)	Na^+^	C_57_H_100_O_6_	0.44	0.007
	653.4408	TG(O-37:9); O_2_	H^+^	C_40_H_60_O_7_	0.41	0.001
	681.4719	TG(O-39:9); O_2_	H^+^	C_42_H_64_O_7_	0.39	0.007
DG						
	563.5029	Mix DG (33:1)	-H_2_O^+^H^+^	C_36_H_68_O_5_	8.01	0.019
	591.5337	Mix DG (35:1)	-H_2_O^+^H^+^	C_38_H_72_O_5_	6.76	0.018
	617.5495	DG (37:2)	-H_2_O^+^H^+^	C_40_H_74_O_5_	2.64	0.038
	641.5107	DG (36:3)	Na^+^	C_39_H_70_O_5_	0.42	0.004
	639.4951	DG (36:4)	Na^+^	C_39_H_68_O_5_	0.41	0.004
SM						
	787.6681	SM (40:1);O_2_	H^+^	C_45_H_91_N_2_O_6_P	5.94	0.015
PC						
	858.5976	PC (40:5)	Na^+^	C_48_H_86_NO_8_P	2.85	0.003
	854.5668	PC (40:7)	Na^+^	C_48_H_82_NO_8_P	0.43	0.003
	860.6144	PC (42:7)	H^+^	C_50_H_86_NO_8_P	0.27	0.004
Dihydro-PC						
	835.6033	DihydroPC (35:0); O_5_	NH_4_^+^	C_40_H_82_NO_13_P	2.46	0.020
Others						
	386.3264	CAR (15:0)	H^+^	C_22_H_43_NO_4_	2.90	0.032
	386.2894	CAR (14:1); O	H^+^	C_21_H_39_NO_5_	0.25	0.006
	440.3369	CAR (18:2); O	H^+^	C_25_H_45_NO_5_	0.24	0.005
	414.3209	CAR (16:1); O	H^+^	C_23_H_43_NO_5_	0.21	0.019
	442.3521	CAR (18:1); O	H^+^	C_25_H_47_NO_5_	0.18	0.019

## Data Availability

The data presented in this study are available in the article and Supplementary Materials. Further inquiries can be directed to the corresponding authors.

## Supplementary Materials

The following supporting information can be downloaded at https://www.mdpi.com/article/10.3390/ijms25052702/s1.
