# Supplementary material for: Lipidomics Reveals Myocardial Lipid Composition in a Murine Model of Insulin Resistance Induced by a High-Fat Diet
Source: Int J Mol Sci. 2024 Feb 26;25(5):2702. doi: 10.3390/ijms25052702 (PMC10932381; doi:10.3390/ijms25052702)
Supplement: Supplementary file 1 [file ijms-25-02702-s001.zip › Supplemental Table S1.pdf]

**Table S1.** Spearman's correlation between the altered myocardial metabolites and weight and biochemical variables related to glucose and fatty acid metabolism. Significant differences are indicated in bold.

| Metabolites | Weight | | Triglycerides | | NEFAs | | Total Cholesterol | | HDLc | | LDLc | | VLDLc | | Glucose | | Insulin | | HOMA-IR | | GTT | | Leptin | | Resistin | | Adiponectin | |
|---|---|---|---|---|---|---|---|---|---|---|---|---|---|---|---|---|---|---|---|---|---|---|---|---|---|---|---|---|
| | r | p | r | P | r | p | r | p | r | p | r | p | r | p | r | p | r | p | r | p | r | p | r | p | r | p | r | p |
| ***Triglycerides*** | | | | | | | | | | | | | | | | | | | | | | | | | | | | |
| Mix TG (49:1) | 0.311 | 0.260 | **0.656** | **0.011** | 0.364 | 0.182 | -0.231 | 0.408 | -0.325 | 0.237 | -0.420 | 0.119 | **0.634** | **0.011** | **0.708** | **0.005** | 0.373 | 0.210 | 0.479 | 0.098 | **0.520** | **0.047** | **0.692** | **0.006** | **0.604** | **0.029** | -0.400 | 0.140 |
| Mix TG (47:1) | 0.375 | 0.168 | **0.673** | **0.008** | 0.318 | 0.248 | -0.354 | 0.196 | -0.481 | 0.070 | -0.474 | 0.074 | **0.631** | **0.012** | **0.702** | **0.005** | 0.267 | 0.378 | 0.374 | 0.208 | 0.488 | 0.065 | **0.736** | **0.003** | **0.440** | **0.133** | **-0.546** | **0.035** |
| Mix TG (51:1) | 0.250 | 0.369 | **0.651** | **0.012** | 0.407 | 0.132 | -0.188 | 0.503 | -0.239 | 0.390 | -0.462 | 0.083 | **0.656** | **0.008** | **0.612** | **0.020** | 0.384 | 0.195 | 0.470 | 0.105 | 0.504 | 0.055 | **0.618** | **0.019** | **0.643** | **0.018** | -0.343 | 0.211 |
| Mix TG (49:2) | 0.250 | 0.369 | **0.616** | **0.019** | 0.425 | 0.114 | -0.188 | 0.503 | -0.261 | 0.348 | -0.408 | 0.131 | **0.613** | **0.015** | **0.605** | **0.022** | 0.317 | 0.291 | 0.429 | 0.143 | 0.427 | 0.112 | **0.644** | **0.013** | **0.555** | **0.049** | -0.461 | 0.084 |
| Mix TG (51:2) | 0.239 | 0.390 | **0.642** | **0.013** | 0.436 | 0.104 | -0.148 | 0.598 | -0.161 | 0.567 | -0.447 | 0.095 | **0.652** | **0.008** | **0.546** | **0.044** | 0.389 | 0.188 | 0.490 | 0.089 | 0.438 | 0.103 | **0.534** | **0.049** | **0.632** | **0.021** | -0.350 | 0.201 |
| Mix TG (42:0) | 0.261 | 0.348 | **0.664** | **0.010** | 0.429 | 0.111 | -0.209 | 0.454 | -0.338 | 0.218 | -0.343 | 0.210 | **0.627** | **0.012** | **0.779** | **0.001** | 0.398 | 0.178 | 0.539 | 0.057 | 0.436 | 0.104 | **0.701** | **0.005** | 0.462 | 0.112 | **-0.521** | **0.046** |
| Mix TG (45:1) | 0.443 | 0.098 | **0.700** | **0.005** | 0.339 | 0.216 | -0.391 | 0.149 | -0.488 | 0.065 | **-0.540** | **0.038** | **0.649** | **0.009** | **0.579** | **0.030** | 0.120 | 0.697 | 0.223 | 0.464 | 0.499 | 0.058 | **0.653** | **0.011** | 0.335 | 0.263 | **-0.643** | **0.010** |
| Mix TG (44:0) | 0.207 | 0.459 | **0.634** | **0.015** | 0.418 | 0.121 | -0.213 | 0.447 | -0.272 | 0.327 | -0.447 | 0.095 | **0.627** | **0.012** | **0.587** | **0.027** | 0.348 | 0.244 | 0.437 | 0.135 | 0.406 | 0.134 | **0.609** | **0.021** | 0.511 | 0.074 | -0.471 | 0.076 |
| TG (53:6) | 0.457 | 0.087 | **0.678** | **0.008** | 0.471 | 0.076 | -0.157 | 0.576 | -0.327 | 0.234 | -0.254 | 0.361 | **0.581** | **0.023** | **0.704** | **0.005** | 0.456 | 0.117 | 0.476 | 0.100 | **0.720** | **0.002** | **0.719** | **0.004** | **0.582** | **0.037** | **-0.593** | **0.020** |
| Mix TG (53:1) | 0.196 | 0.483 | **0.687** | **0.007** | 0.382 | 0.160 | -0.225 | 0.420 | -0.143 | 0.611 | **-0.589** | **0.021** | **0.728** | **0.002** | 0.444 | 0.111 | **0.573** | **0.041** | **0.622** | **0.023** | 0.270 | 0.331 | 0.345 | 0.227 | 0.423 | 0.150 | -0.296 | 0.283 |
| Mix TG (53:2) | 0.179 | 0.524 | **0.559** | **0.038** | 0.293 | 0.289 | -0.181 | 0.520 | -0.172 | 0.541 | -0.433 | 0.107 | **0.584** | **0.022** | 0.464 | 0.094 | 0.426 | 0.147 | 0.514 | 0.072 | 0.384 | 0.157 | 0.451 | 0.106 | 0.544 | 0.055 | -0.229 | 0.413 |
| Mix TG (O-51:4); O | 0.211 | 0.451 | **0.559** | **0.038** | 0.425 | 0.114 | -0.148 | 0.598 | -0.154 | 0.584 | -0.408 | 0.131 | **0.581** | **0.023** | 0.510 | 0.062 | 0.353 | 0.236 | 0.459 | 0.114 | 0.395 | 0.145 | 0.516 | 0.059 | **0.599** | **0.031** | -0.346 | 0.206 |
| Mix TG (O-52:8) | 0.482 | 0.069 | **0.589** | **0.027** | 0.382 | 0.160 | -0.272 | 0.327 | -0.395 | 0.145 | -0.386 | 0.155 | **0.548** | **0.034** | **0.713** | **0.004** | 0.278 | 0.357 | 0.377 | 0.204 | **0.520** | **0.047** | **0.692** | **0.006** | 0.434 | 0.138 | **-0.600** | **0.018** |
| Mix TG (47:0) | 0.307 | 0.265 | **0.687** | **0.007** | 0.418 | 0.121 | -0.270 | 0.331 | -0.406 | 0.134 | -0.403 | 0.137 | **0.642** | **0.010** | **0.779** | **0.001** | 0.495 | 0.085 | **0.575** | **0.040** | **0.527** | **0.043** | **0.705** | **0.005** | 0.473 | 0.103 | **-0.532** | **0.041** |
| Mix TG (40:0) | 0.275 | 0.321 | **0.726** | **0.003** | 0.329 | 0.232 | -0.332 | 0.226 | -0.447 | 0.095 | -0.463 | 0.082 | **0.685** | **0.005** | **0.735** | **0.003** | 0.351 | 0.240 | 0.476 | 0.100 | 0.408 | 0.132 | **0.697** | **0.006** | 0.429 | 0.144 | -0.500 | 0.058 |
| Mix TG (O-53:4); O | 0.111 | 0.694 | 0.408 | 0.147 | 0.325 | 0.237 | -0.107 | 0.704 | -0.070 | 0.805 | -0.351 | 0.200 | 0.477 | 0.072 | 0.301 | 0.295 | 0.348 | 0.244 | 0.454 | 0.119 | 0.349 | 0.203 | 0.367 | 0.197 | 0.495 | 0.086 | -0.236 | 0.398 |
| Mix TG (46:0) | 0.082 | 0.771 | 0.514 | 0.060 | 0.332 | 0.226 | -0.163 | 0.562 | -0.175 | 0.532 | -0.408 | 0.131 | **0.548** | **0.034** | 0.420 | 0.135 | 0.339 | 0.257 | 0.415 | 0.158 | 0.306 | 0.268 | 0.481 | 0.081 | 0.440 | 0.133 | -0.343 | 0.211 |
| Mix TG (45:2) | 0.339 | 0.216 | **0.589** | **0.027** | **0.525** | **0.044** | -0.179 | 0.524 | -0.231 | 0.408 | -0.397 | 0.143 | **0.566** | **0.028** | 0.486 | 0.078 | 0.186 | 0.542 | 0.305 | 0.310 | 0.438 | 0.103 | **0.534** | **0.049** | 0.467 | 0.108 | **-0.661** | **0.007** |
| Mix TG (49:0) | 0.250 | 0.369 | **0.700** | **0.005** | 0.418 | 0.121 | -0.273 | 0.324 | -0.365 | 0.181 | -0.442 | 0.099 | **0.685** | **0.005** | **0.669** | **0.009** | 0.512 | 0.074 | **0.558** | **0.047** | 0.415 | 0.124 | **0.626** | **0.017** | 0.478 | 0.098 | -0.504 | 0.056 |
| Mix TG (43:0) | 0.311 | 0.260 | **0.642** | **0.013** | 0.486 | 0.066 | -0.177 | 0.528 | -0.189 | 0.499 | -0.479 | 0.071 | **0.638** | **0.010** | 0.482 | 0.081 | 0.334 | 0.265 | 0.413 | 0.161 | 0.388 | 0.153 | 0.407 | 0.149 | 0.346 | 0.247 | **-0.614** | **0.015** |
| TG(O-54:7) | 0.336 | 0.221 | **0.722** | **0.004** | 0.336 | 0.221 | -0.306 | 0.268 | -0.354 | 0.196 | **-0.526** | **0.044** | **0.695** | **0.004** | **0.667** | **0.009** | 0.451 | 0.122 | 0.517 | 0.070 | **0.545** | **0.036** | **0.574** | **0.032** | **0.582** | **0.037** | -0.393 | 0.147 |
| Mix TG (55:6) | 0.400 | 0.140 | **0.642** | **0.013** | **0.543** | **0.037** | -0.197 | 0.482 | -0.347 | 0.205 | -0.297 | 0.282 | **0.595** | **0.019** | **0.658** | **0.011** | 0.353 | 0.236 | 0.421 | 0.152 | **0.620** | **0.014** | **0.684** | **0.007** | 0.500 | 0.082 | **-0.732** | **0.002** |
| Mix TG (55:7) | 0.500 | 0.058 | **0.669** | **0.009** | 0.454 | 0.089 | -0.163 | 0.562 | -0.352 | 0.198 | -0.301 | 0.276 | **0.581** | **0.023** | **0.625** | **0.017** | 0.253 | 0.404 | 0.286 | 0.343 | **0.767** | **0.001** | **0.802** | **0.001** | **0.615** | **0.025** | **-0.561** | **0.030** |
| Mix TG (45:0) | 0.411 | 0.128 | **0.682** | **0.007** | 0.339 | 0.216 | -0.338 | 0.218 | -0.393 | 0.147 | -0.510 | 0.052 | **0.627** | **0.012** | **0.700** | **0.005** | 0.442 | 0.130 | 0.492 | 0.087 | **0.577** | **0.024** | **0.604** | **0.022** | 0.533 | 0.061 | -0.439 | 0.101 |
| Mix TG (O-54:6); O | 0.221 | 0.428 | **0.638** | **0.014** | 0.329 | 0.232 | -0.243 | 0.383 | -0.263 | 0.344 | -0.485 | 0.067 | **0.642** | **0.010** | **0.598** | **0.024** | 0.456 | 0.117 | 0.523 | 0.067 | 0.449 | 0.093 | **0.538** | **0.047** | **0.577** | **0.039** | -0.300 | 0.277 |
| Mix TG (55:2) | 0.154 | 0.585 | 0.519 | 0.057 | 0.311 | 0.260 | -0.152 | 0.589 | -0.129 | 0.648 | -0.424 | 0.115 | **0.552** | **0.033** | 0.385 | 0.174 | 0.431 | 0.141 | 0.501 | 0.081 | 0.429 | 0.111 | 0.402 | 0.154 | **0.560** | **0.046** | -0.204 | 0.467 |
| Mix TG (O-55:7); O | 0.314 | 0.254 | 0.475 | 0.086 | 0.511 | 0.052 | 0.120 | 0.671 | -0.032 | 0.909 | -0.075 | 0.790 | 0.437 | 0.103 | 0.519 | 0.057 | 0.412 | 0.162 | 0.484 | 0.094 | **0.744** | **0.001** | **0.635** | **0.015** | **0.588** | **0.035** | -0.379 | 0.164 |
| Mix TG (O-55:4); O | 0.154 | 0.585 | 0.430 | 0.124 | 0.250 | 0.369 | -0.091 | 0.747 | -0.043 | 0.879 | -0.422 | 0.117 | 0.495 | 0.061 | 0.235 | 0.418 | 0.409 | 0.165 | 0.457 | 0.117 | 0.391 | 0.149 | 0.297 | 0.303 | 0.429 | 0.144 | -0.154 | 0.585 |
| Mix TG (53:3) | 0.232 | 0.405 | 0.351 | 0.219 | 0.329 | 0.232 | -0.045 | 0.874 | -0.043 | 0.879 | -0.279 | 0.314 | 0.401 | 0.138 | 0.363 | 0.202 | 0.303 | 0.314 | 0.391 | 0.187 | 0.393 | 0.147 | 0.429 | 0.126 | 0.484 | 0.094 | -0.236 | 0.398 |
| Mix TG (57:2) | 0.204 | 0.467 | 0.457 | 0.100 | 0.293 | 0.289 | -0.123 | 0.661 | -0.116 | 0.680 | -0.388 | 0.153 | 0.487 | 0.065 | 0.392 | 0.166 | 0.465 | 0.110 | 0.492 | 0.087 | 0.492 | 0.063 | 0.420 | 0.135 | **0.555** | **0.049** | -0.154 | 0.585 |
| Mix TG (O-55:6); O | 0.093 | 0.742 | 0.369 | 0.195 | 0.436 | 0.104 | 0.045 | 0.874 | 0.057 | 0.840 | -0.222 | 0.427 | 0.466 | 0.080 | 0.185 | 0.527 | 0.420 | 0.153 | 0.476 | 0.100 | 0.395 | 0.145 | 0.363 | 0.203 | 0.473 | 0.103 | -0.264 | 0.341 |
| Mix TG (O-49:1); O | 0.354 | 0.196 | **0.709** | **0.005** | 0.304 | 0.271 | -0.415 | 0.124 | **-0.531** | **0.042** | -0.465 | 0.081 | **0.667** | **0.007** | **0.704** | **0.005** | 0.426 | 0.147 | 0.451 | 0.122 | 0.436 | 0.104 | **0.719** | **0.004** | 0.489 | 0.090 | **-0.546** | **0.035** |
| Mix TG (54:6) | 0.279 | 0.315 | **0.603** | **0.023** | 0.261 | 0.348 | -0.189 | 0.499 | -0.284 | 0.305 | -0.352 | 0.198 | **0.570** | **0.027** | 0.453 | 0.104 | 0.453 | 0.120 | 0.421 | 0.152 | **0.615** | **0.015** | **0.596** | **0.025** | **0.588** | **0.035** | -0.254 | 0.362 |
| Mix TG (51:0) | 0.229 | 0.413 | **0.620** | **0.018** | 0.375 | 0.168 | -0.211 | 0.451 | -0.282 | 0.308 | -0.376 | 0.168 | **0.602** | **0.018** | **0.653** | **0.011** | 0.434 | 0.138 | 0.476 | 0.100 | 0.384 | 0.157 | **0.635** | **0.015** | 0.473 | 0.103 | -0.404 | 0.136 |
| Mix TG (O-54:6) | 0.275 | 0.321 | 0.510 | 0.062 | 0.271 | 0.328 | -0.166 | 0.554 | -0.270 | 0.331 | -0.295 | 0.286 | 0.495 | 0.061 | 0.425 | 0.130 | 0.417 | 0.156 | 0.399 | 0.177 | **0.617** | **0.014** | **0.609** | **0.021** | **0.626** | **0.022** | -0.232 | 0.405 |
| Mix TG (38:0) | 0.368 | 0.177 | **0.779** | **0.001** | 0.461 | 0.084 | -0.306 | 0.268 | -0.331 | 0.229 | -0.499 | 0.058 | **0.738** | **0.002** | **0.730** | **0.003** | **0.609** | **0.027** | **0.710** | **0.007** | 0.361 | 0.186 | 0.508 | 0.064 | 0.368 | 0.216 | **-0.521** | **0.046** |
| Mix TG (O-57:8); O | 0.293 | 0.289 | **0.581** | **0.029** | 0.425 | 0.114 | -0.039 | 0.889 | -0.220 | 0.431 | -0.166 | 0.553 | **0.520** | **0.047** | **0.640** | **0.014** | 0.478 | 0.098 | 0.550 | 0.051 | **0.711** | **0.003** | **0.758** | **0.002** | **0.632** | **0.021** | -0.314 | 0.254 |
| Mix TG (O-53:7) | 0.107 | 0.704 | **0.541** | **0.046** | 0.346 | 0.206 | -0.306 | 0.268 | -0.322 | 0.242 | -0.503 | 0.056 | **0.556** | **0.032** | **0.532** | **0.050** | 0.423 | 0.150 | 0.473 | 0.102 | 0.320 | 0.245 | 0.508 | 0.064 | 0.473 | 0.103 | -0.339 | 0.216 |
| Mix TG (O-55:8) | 0.404 | 0.136 | **0.656** | **0.011** | 0.407 | 0.132 | -0.181 | 0.520 | -0.286 | 0.301 | -0.340 | 0.215 | **0.642** | **0.010** | **0.561** | **0.037** | 0.242 | 0.426 | 0.300 | 0.320 | 0.384 | 0.157 | **0.679** | **0.008** | 0.544 | 0.055 | **-0.543** | **0.037** |
| Mix TG (60:9) | -0.379 | 0.164 | **-0.753** | **0.002** | -0.264 | 0.341 | 0.275 | 0.321 | 0.324 | 0.240 | 0.501 | 0.057 | **-0.656** | **0.008** | **-0.801** | **0.001** | -0.317 | 0.291 | -0.454 | 0.119 | -0.263 | 0.344 | -0.437 | 0.118 | -0.104 | 0.734 | 0.432 | 0.108 |
| Mix TG (O-55:11); O | -0.471 | 0.076 | **-0.788** | **0.001** | -0.164 | 0.558 | 0.313 | 0.256 | 0.492 | 0.063 | 0.224 | 0.423 | -0.491 | 0.063 | **-0.704** | **0.005** | -0.348 | 0.244 | -0.426 | 0.146 | -0.254 | 0.361 | -0.530 | 0.051 | -0.005 | 0.986 | **0.596** | **0.019** |
| Mix TG (O-59:11); O | -0.400 | 0.140 | **-0.762** | **0.002** | -0.232 | 0.405 | 0.238 | 0.394 | 0.365 | 0.181 | 0.274 | 0.324 | -0.513 | 0.051 | **-0.832** | **<0.001** | -0.428 | 0.144 | **-0.586** | **0.035** | -0.277 | 0.318 | -0.521 | 0.056 | -0.121 | 0.694 | 0.432 | 0.108 |
| Mix TG (54:4) | -0.382 | 0.160 | **-0.806** | **0.001** | -0.246 | 0.376 | 0.275 | 0.321 | 0.441 | 0.099 | 0.290 | 0.295 | -0.509 | 0.053 | **-0.849** | **<0.001** | -0.456 | 0.117 | **-0.608** | **0.027** | -0.363 | 0.184 | **-0.604** | **0.022** | -0.165 | 0.590 | 0.486 | 0.066 |
| Mix TG (58:6) | -0.400 | 0.140 | **-0.762** | **0.002** | -0.232 | 0.405 | 0.238 | 0.394 | 0.365 | 0.181 | 0.274 | 0.324 | -0.513 | 0.051 | **-0.832** | **<0.001** | -0.428 | 0.144 | **-0.586** | **0.035** | -0.277 | 0.318 | -0.521 | 0.056 | -0.121 | 0.694 | 0.432 | 0.108 |
| Mix TG (55:3); O | -0.446 | 0.095 | **-0.691** | **0.006** | -0.046 | 0.869 | 0.472 | 0.076 | **0.559** | **0.030** | 0.458 | 0.086 | -0.495 | 0.061 | **-0.761** | **0.002** | -0.253 | 0.404 | -0.344 | 0.250 | -0.195 | 0.487 | -0.429 | 0.126 | -0.016 | 0.957 | 0.443 | 0.098 |
| Mix TG (54:5) | -0.482 | 0.069 | **-0.841** | **<0.001** | -0.293 | 0.289 | 0.307 | 0.265 | 0.484 | 0.067 | 0.306 | 0.268 | **-0.534** | **0.040** | **-0.832** | **<0.001** | -0.476 | 0.100 | **-0.586** | **0.035** | -0.424 | **0.116** | **-0.613** | 0.020 | -0.181 | 0.553 | **0.582** | **0.023** |
| TG(O-37:9); O2 | **-0.761** | **0.001** | **-0.841** | **<0.001** | -0.496 | 0.060 | 0.111 | 0.694 | 0.290 | 0.295 | 0.252 | 0.364 | **-0.685** | **0.005** | **-0.722** | **0.004** | -0.465 | 0.110 | -0.495 | 0.085 | **-0.617** | **0.014** | **-0.657** | **0.011** | -0.440 | 0.133 | **0.546** | **0.035** |
| TG(O-39:9); O2 | **-0.711** | **0.003** | **-0.740** | **0.003** | -0.421 | 0.118 | 0.093 | 0.742 | 0.340 | 0.216 | 0.041 | 0.884 | -0.444 | 0.097 | **-0.733** | **0.003** | -0.437 | 0.136 | -0.498 | 0.083 | **-0.574** | **0.025** | **-0.705** | **0.005** | -0.286 | 0.344 | **0.589** | **0.021** |
| ***Diacylglycerols*** | | | | | | | | | | | | | | | | | | | | | | | | | | | | |
| Mix DG (33:1) | 0.279 | 0.315 | **0.687** | **0.007** | 0.379 | 0.164 | -0.241 | 0.386 | -0.263 | 0.344 | -0.503 | 0.056 | **0.695** | **0.004** | **0.579** | **0.030** | 0.428 | 0.144 | 0.517 | 0.070 | 0.459 | **0.085** | **0.547** | **0.043** | **0.632** | **0.021** | -0.350 | 0.201 |
| Mix DG (35:1) | 0.232 | 0.405 | **0.603** | **0.023** | 0.325 | 0.237 | -0.220 | 0.431 | -0.197 | 0.482 | -0.478 | 0.072 | **0.620** | **0.014** | 0.484 | 0.079 | 0.445 | 0.127 | 0.528 | 0.064 | 0.406 | 0.134 | 0.437 | 0.118 | **0.588** | **0.035** | -0.257 | 0.355 |
| DG (37:2) | 0.161 | 0.567 | 0.404 | 0.152 | 0.321 | 0.243 | -0.025 | 0.929 | 0.050 | 0.859 | -0.401 | 0.139 | 0.487 | 0.065 | 0.183 | 0.532 | 0.381 | 0.199 | 0.424 | 0.149 | 0.377 | 0.166 | 0.253 | 0.383 | 0.401 | 0.174 | -0.186 | 0.508 |
| DG (36:3) | -0.489 | 0.064 | **-0.740** | **0.003** | -0.257 | 0.355 | 0.286 | 0.301 | 0.425 | 0.114 | 0.385 | 0.157 | **-0.566** | **0.028** | **-0.700** | **0.005** | -0.053 | 0.864 | -0.215 | 0.481 | -0.361 | 0.186 | **-0.574** | **0.032** | -0.110 | 0.721 | **0.575** | **0.025** |
| DG (36:4) | **-0.518** | **0.048** | **-0.726** | **0.003** | -0.364 | 0.182 | 0.216 | 0.439 | 0.331 | 0.229 | 0.444 | 0.098 | **-0.634** | **0.011** | **-0.697** | **0.006** | -0.070 | 0.821 | -0.215 | 0.481 | -0.402 | 0.137 | **-0.560** | **0.037** | -0.192 | 0.529 | **0.607** | **0.016** |
| ***Sphingomyelins*** | | | | | | | | | | | | | | | | | | | | | | | | | | | | |
| SM (40:1);O2 | 0.418 | 0.121 | **0.660** | **0.010** | 0.375 | 0.168 | -0.356 | 0.193 | -0.466 | 0.080 | -0.478 | 0.072 | **0.616** | **0.014** | **0.620** | **0.018** | 0.164 | 0.592 | 0.278 | 0.358 | 0.509 | 0.052 | **0.684** | **0.007** | 0.412 | 0.162 | **-0.639** | **0.010** |
| ***Phosphatidylcholines*** | | | | | | | | | | | | | | | | | | | | | | | | | | | | |
| PC (40:5) | 0.446 | 0.095 | 0.457 | 0.100 | 0.425 | 0.114 | -0.100 | 0.723 | -0.263 | 0.344 | -0.179 | 0.524 | 0.355 | 0.194 | 0.524 | 0.055 | 0.289 | 0.338 | 0.330 | 0.271 | **0.747** | **0.001** | **0.662** | **0.010** | 0.549 | 0.052 | **-0.518** | **0.048** |
| PC (40:7) | **-0.664** | **0.007** | **-0.638** | **0.014** | -0.143 | 0.612 | 0.279 | 0.314 | 0.384 | 0.157 | 0.372 | 0.172 | -0.509 | 0.053 | **-0.563** | **0.036** | -0.209 | 0.494 | -0.206 | 0.499 | -0.315 | 0.253 | -0.512 | 0.061 | -0.203 | 0.505 | 0.279 | 0.315 |
| PC (42:7) | **-0.643** | **0.010** | **-0.845** | **<0.001** | -0.429 | 0.111 | 0.282 | 0.308 | 0.391 | 0.149 | 0.478 | 0.072 | **-0.763** | **0.001** | **-0.739** | **0.003** | -0.381 | 0.199 | -0.432 | 0.141 | -0.463 | 0.082 | **-0.635** | **0.015** | -0.379 | 0.201 | **0.575** | **0.025** |
| ***Dihydro-Phosphatidylcholines*** | | | | | | | | | | | | | | | | | | | | | | | | | | | | |
| DihydroPC (35:0); O5 | 0.204 | 0.467 | 0.272 | 0.348 | 0.418 | 0.121 | 0.025 | 0.929 | -0.118 | 0.675 | -0.047 | 0.869 | 0.201 | 0.473 | 0.348 | 0.223 | 0.392 | 0.185 | 0.404 | 0.171 | **0.667** | **0.007** | 0.477 | 0.085 | 0.445 | 0.128 | -0.414 | 0.125 |
| ***Carnitines*** | | | | | | | | | | | | | | | | | | | | | | | | | | | | |
| CAR (15:0) | 0.339 | 0.216 | **0.647** | **0.012** | 0.189 | 0.499 | -0.316 | 0.251 | -0.511 | 0.051 | -0.197 | 0.482 | 0.459 | 0.085 | 0.592 | 0.026 | 0.220 | 0.471 | 0.303 | 0.315 | **0.579** | **0.024** | **0.692** | **0.006** | 0.341 | 0.255 | **-0.568** | **0.027** |
| CAR (14:1); O | **-0.714** | **0.003** | **-0.757** | **0.002** | -0.368 | 0.177 | 0.139 | 0.620 | 0.320 | 0.245 | 0.313 | 0.256 | **-0.581** | **0.023** | **-0.682** | **0.007** | -0.172 | 0.573 | -0.253 | 0.404 | **-0.550** | **0.033** | **-0.675** | **0.008** | -0.297 | 0.325 | **0.546** | **0.035** |
| CAR (18:2); O | -0.511 | 0.052 | **-0.757** | **0.002** | -0.436 | 0.104 | 0.107 | 0.704 | 0.241 | 0.386 | 0.345 | 0.208 | **-0.660** | **0.007** | **-0.792** | **0.001** | -0.378 | 0.202 | -0.512 | 0.074 | -0.461 | 0.084 | **-0.662** | **0.010** | -0.346 | 0.247 | 0.504 | 0.056 |
| CAR (16:1); O | -0.493 | 0.062 | **-0.766** | **0.001** | -0.321 | 0.243 | 0.097 | 0.732 | 0.263 | 0.344 | 0.309 | 0.262 | **-0.595** | **0.019** | **-0.792** | **0.001** | -0.167 | 0.586 | -0.330 | 0.271 | -0.422 | 0.117 | **-0.609** | **0.021** | -0.159 | 0.603 | 0.504 | 0.056 |
| CAR (18:1); O | -0.471 | 0.076 | **-0.731** | **0.003** | -0.382 | 0.160 | 0.063 | 0.825 | 0.213 | 0.447 | 0.317 | 0.250 | **-0.595** | **0.019** | **-0.752** | **0.002** | -0.134 | 0.664 | -0.308 | 0.306 | -0.447 | 0.095 | **-0.618** | **0.019** | -0.214 | 0.482 | 0.500 | 0.058 |
