# Supplementary material for: Lipidomics Reveals Myocardial Lipid Composition in a Murine Model of Insulin Resistance Induced by a High-Fat Diet
Source: Int J Mol Sci. 2024 Feb 26;25(5):2702. doi: 10.3390/ijms25052702 (PMC10932381; doi:10.3390/ijms25052702)
Supplement: Supplementary file 1 [file ijms-25-02702-s001.zip › Supplemental Table S2.pdf]

Table S2. Spearman's correlation between the altered myocardial metabolites and liver TG. Significant differences are indicated in bold.

Metabolites	TG(51:2)		TG(51:1)		TG(49:1)		TG(53:1)		TG(46:0)		TG(49:2)		TG(49:0)		TG(44:1)		TG(47:0)		TG(44:0)		TG(43:1)		TG(48:1)		TG(52:5)		TG(48:0)	
	r	*p*	*r*	*P*	*r*	*p*	*r*	*p*	*r*	*p*	*r*	*P*	*r*	*p*	*r*	*p*	*r*	*p*	*r*	*p*	*r*	*p*	*r*	*p*	*r*	*p*	*r*	*p*
Triglycerides																												
Mix TG (49:1)	**0.689**	**0.004**	**0.654**	**0.008**	**0.668**	**0.007**	**0.589**	**0.021**	**0.704**	**0.003**	**0.546**	**0.035**	**0.654**	**0.008**	0.411	0.128	**0.675**	**0.006**	**0.564**	**0.028**	0.332	0.226	0.432	0.108	0.389	0.152	**0.646**	**0.009**
Mix TG (47:1)	**0.714**	**0.003**	**0.704**	**0.003**	**0.700**	**0.004**	**0.700**	**0.004**	**0.761**	**0.001**	**0.561**	**0.030**	**0.718**	**0.003**	0.436	0.104	**0.696**	**0.004**	**0.646**	**0.009**	0.282	0.308	**0.532**	**0.041**	0.418	0.121	**0.614**	**0.015**
Mix TG (51:1)	**0.664**	**0.007**	**0.589**	**0.021**	**0.611**	**0.016**	**0.518**	**0.048**	**0.679**	**0.005**	0.504	0.056	**0.611**	**0.016**	0.389	0.152	**0.643**	**0.010**	**0.539**	**0.038**	0.325	0.237	0.407	0.132	0.357	0.191	**0.657**	**0.008**
Mix TG (49:2)	**0.650**	**0.009**	**0.614**	**0.015**	**0.632**	**0.011**	**0.550**	**0.034**	**0.671**	**0.006**	0.486	0.066	**0.579**	**0.024**	0.400	0.140	**0.579**	**0.024**	0.507	0.054	0.314	0.254	0.414	0.125	0.329	0.232	**0.586**	**0.022**
Mix TG (51:2)	**0.611**	**0.016**	**0.529**	**0.043**	**0.564**	**0.028**	**0.461**	0.084	**0.646**	**0.009**	0.471	0.076	**0.561**	**0.030**	0.386	0.156	**0.579**	**0.024**	0.493	0.062	0.343	0.211	0.361	0.187	0.321	0.243	**0.650**	**0.009**
Mix TG (42:0)	**0.679**	**0.005**	**0.707**	**0.003**	**0.711**	**0.003**	**0.643**	**0.010**	**0.661**	**0.007**	0.504	0.056	**0.618**	**0.014**	0.357	0.191	**0.621**	**0.013**	0.482	0.069	0.243	0.383	0.382	0.160	0.339	0.216	**0.539**	**0.038**
Mix TG (45:1)	**0.750**	**0.001**	**0.682**	**0.005**	**0.682**	**0.005**	**0.754**	**0.001**	**0.779**	**0.001**	**0.582**	**0.023**	**0.793**	**<0.001**	0.457	0.087	**0.736**	**0.002**	**0.714**	**0.003**	0.271	0.328	**0.582**	**0.023**	0.489	0.064	**0.629**	**0.012**
Mix TG (44:0)	**0.661**	**0.007**	**0.618**	**0.014**	**0.629**	**0.012**	**0.561**	**0.030**	**0.675**	**0.006**	0.471	0.076	**0.589**	**0.021**	0.371	0.173	**0.582**	**0.023**	0.493	0.062	0.279	0.315	0.404	0.136	0.311	0.260	**0.614**	**0.015**
TG (53:6)	**0.889**	**<0.001**	**0.800**	**<0.001**	**0.782**	**0.001**	**0.768**	**0.001**	**0.825**	**<0.001**	**0.725**	**0.002**	**0.846**	**<0.001**	**0.575**	**0.025**	**0.836**	**<0.001**	**0.700**	**0.004**	0.461	0.084	**0.650**	**0.009**	**0.561**	**0.030**	**0.836**	**<0.001**
Mix TG (53:1)	0.450	0.092	0.396	0.143	0.432	0.108	0.379	0.164	**0.643**	**0.010**	0.382	0.160	0.511	0.052	0.343	0.211	0.504	0.056	0.482	0.069	0.279	0.315	0.346	0.206	0.214	0.443	**0.711**	**0.003**
Mix TG (53:2)	**0.529**	**0.043**	0.443	0.098	0.468	0.079	0.379	0.164	**0.582**	**0.023**	0.418	0.121	**0.514**	**0.050**	0.321	0.243	**0.536**	**0.040**	0.450	0.092	0.318	0.248	0.329	0.232	0.282	0.308	**0.646**	**0.009**
Mix TG (O-51:4); O	**0.596**	**0.019**	**0.514**	**0.050**	**0.557**	**0.031**	0.439	0.101	**0.593**	**0.020**	0.439	0.101	**0.518**	**0.048**	0.343	0.211	**0.536**	**0.040**	0.436	0.104	0.318	0.248	0.311	0.260	0.311	0.260	**0.596**	**0.019**
Mix TG (O-52:8)	**0.721**	**0.002**	**0.711**	**0.003**	**0.729**	**0.002**	**0.693**	**0.004**	**0.800**	**<0.001**	**0.614**	**0.015**	**0.700**	**0.004**	**0.536**	**0.040**	**0.668**	**0.007**	**0.668**	**0.007**	0.379	0.164	**0.589**	**0.021**	0.443	0.098	**0.668**	**0.007**
Mix TG (47:0)	**0.732**	**0.002**	**0.725**	**0.002**	**0.714**	**0.003**	**0.679**	**0.005**	**0.764**	**0.001**	**0.546**	**0.035**	**0.707**	**0.003**	0.421	0.118	**0.700**	**0.004**	**0.604**	**0.017**	0.268	0.334	**0.529**	**0.043**	0.361	0.187	**0.686**	**0.005**
Mix TG (40:0)	**0.654**	**0.008**	**0.671**	**0.006**	**0.664**	**0.007**	**0.650**	**0.009**	**0.689**	**0.004**	0.489	0.064	**0.654**	**0.008**	0.343	0.211	**0.646**	**0.009**	**0.546**	**0.035**	0.207	0.459	0.414	0.125	0.343	0.211	**0.557**	**0.031**
Mix TG (O-53:4); O	**0.529**	**0.043**	0.382	0.160	0.418	0.121	0.325	0.237	0.464	0.081	0.361	0.187	0.457	0.087	0.246	0.376	0.471	0.076	0.350	0.201	0.282	0.308	0.243	0.383	0.289	0.296	**0.557**	**0.031**
Mix TG (46:0)	**0.564**	**0.028**	0.493	0.062	0.500	0.058	0.421	0.118	**0.557**	**0.031**	0.379	0.164	0.468	0.079	0.282	0.308	0.471	0.076	0.375	0.168	0.250	0.369	0.307	0.265	0.246	0.376	**0.564**	**0.028**
Mix TG (45:2)	**0.682**	**0.005**	**0.582**	**0.023**	**0.607**	**0.016**	**0.582**	**0.023**	**0.729**	**0.002**	0.482	0.069	**0.621**	**0.013**	0.461	0.084	**0.554**	**0.032**	**0.579**	**0.024**	0.296	0.283	**0.536**	**0.040**	0.329	0.232	**0.650**	**0.009**
Mix TG (49:0)	**0.650**	**0.009**	**0.625**	**0.013**	**0.614**	**0.015**	**0.579**	**0.024**	**0.743**	**0.002**	0.468	0.079	**0.614**	**0.015**	0.393	0.147	**0.604**	**0.017**	**0.561**	**0.030**	0.239	0.390	0.489	0.064	0.279	0.315	**0.693**	**0.004**
Mix TG (43:0)	**0.621**	**0.013**	**0.568**	**0.027**	**0.579**	**0.024**	**0.571**	**0.026**	**0.793**	**<0.001**	0.504	0.056	**0.632**	**0.011**	**0.514**	**0.050**	**0.554**	**0.032**	**0.600**	**0.018**	0.354	0.196	**0.586**	**0.022**	0.282	0.308	**0.775**	**0.001**
TG(O-54:7)	**0.682**	**0.005**	**0.586**	**0.022**	**0.596**	**0.019**	**0.568**	**0.027**	**0.757**	**0.001**	**0.529**	**0.043**	**0.714**	**0.003**	0.396	0.143	**0.714**	**0.003**	**0.636**	**0.011**	0.279	0.315	0.482	0.069	0.364	0.182	**0.793**	**<0.001**
Mix TG (55:6)	**0.854**	**<0.001**	**0.764**	**0.001**	**0.750**	**0.001**	**0.761**	**0.001**	**0.818**	**<0.001**	**0.625**	**0.013**	**0.811**	**<0.001**	**0.521**	**0.046**	**0.750**	**0.001**	**0.668**	**0.007**	0.346	0.206	**0.643**	**0.010**	0.450	0.092	**0.782**	**0.001**
Mix TG (55:7)	**0.921**	**<0.001**	**0.825**	**<0.001**	**0.807**	**<0.001**	**0.807**	**<0.001**	**0.829**	**<0.001**	**0.775**	**0.001**	**0.871**	**<0.001**	**0.632**	**0.011**	**0.882**	**<0.001**	**0.771**	**0.001**	**0.529**	**0.043**	**0.707**	**0.003**	**0.668**	**0.007**	**0.743**	**0.002**
Mix TG (45:0)	**0.768**	**0.001**	**0.679**	**0.005**	**0.707**	**0.003**	**0.675**	**0.006**	**0.736**	**0.002**	**0.607**	**0.016**	**0.779**	**0.001**	0.400	0.140	**0.786**	**0.001**	**0.632**	**0.011**	0.307	0.265	0.439	0.101	0.496	0.060	**0.743**	**0.002**
Mix TG (O-54:6); O	**0.625**	**0.013**	**0.550**	**0.034**	**0.571**	**0.026**	0.489	0.064	**0.650**	**0.009**	0.475	0.074	**0.604**	**0.017**	0.332	0.226	**0.629**	**0.012**	0.500	0.058	0.289	0.296	0.350	0.201	0.329	0.232	**0.693**	**0.004**
Mix TG (55:2)	**0.550**	**0.034**	0.404	0.136	0.429	0.111	0.346	0.206	**0.557**	**0.031**	0.396	0.143	**0.518**	**0.048**	0.293	0.289	**0.543**	**0.037**	0.450	0.092	0.289	0.296	0.336	0.221	0.289	0.296	**0.664**	**0.007**
Mix TG (O-55:7); O	**0.814**	**<0.001**	**0.696**	**0.004**	**0.689**	**0.004**	**0.604**	**0.017**	**0.629**	**0.012**	**0.671**	**0.006**	**0.696**	**0.004**	**0.529**	**0.043**	**0.743**	**0.002**	**0.550**	**0.034**	**0.532**	**0.041**	**0.550**	**0.034**	**0.568**	**0.027**	**0.661**	**0.007**
Mix TG (O-55:4); O	0.507	0.054	0.371	0.173	0.389	0.152	0.325	0.237	**0.550**	**0.034**	0.432	0.108	0.489	0.064	0.364	0.182	0.511	0.052	0.457	0.087	0.396	0.143	0.389	0.152	0.332	0.226	**0.661**	**0.007**
Mix TG (53:3)	**0.546**	**0.035**	0.446	0.095	0.493	0.062	0.357	0.191	**0.514**	**0.050**	0.457	0.087	0.436	0.104	0.375	0.168	0.471	0.076	0.396	0.143	0.414	0.125	0.318	0.248	0.368	0.177	**0.546**	**0.035**
Mix TG (57:2)	**0.596**	**0.019**	0.464	0.081	0.489	0.064	0.389	0.152	**0.579**	**0.024**	0.486	0.066	**0.550**	**0.034**	0.368	0.177	**0.600**	**0.018**	0.486	0.066	0.400	0.140	0.389	0.152	0.389	0.152	**0.689**	**0.004**
Mix TG (O-55:6); O	**0.525**	**0.044**	0.379	0.164	0.386	0.156	0.321	0.243	0.489	0.064	0.393	0.147	0.471	0.076	0.354	0.196	0.475	0.074	0.389	0.152	0.389	0.152	0.393	0.147	0.300	0.277	**0.614**	**0.015**
Mix TG (O-49:1); O	**0.714**	**0.003**	**0.689**	**0.004**	**0.675**	**0.006**	**0.679**	**0.005**	**0.775**	**0.001**	**0.543**	**0.037**	**0.711**	**0.003**	0.407	0.132	**0.689**	**0.004**	**0.621**	**0.013**	0.261	0.348	0.500	0.058	0.386	0.156	**0.714**	**0.003**
Mix TG (54:6)	**0.736**	**0.002**	**0.600**	**0.018**	**0.575**	**0.025**	**0.561**	**0.030**	**0.661**	**0.007**	**0.600**	**0.018**	**0.714**	**0.003**	0.429	0.111	**0.761**	**0.001**	**0.604**	**0.017**	0.421	0.118	0.504	0.056	**0.518**	**0.048**	**0.739**	**0.002**
Mix TG (51:0)	**0.693**	**0.004**	**0.689**	**0.004**	**0.704**	**0.003**	**0.614**	**0.015**	**0.600**	**0.018**	**0.539**	**0.038**	**0.593**	**0.020**	0.339	0.216	**0.632**	**0.011**	0.418	0.121	0.321	0.243	0.275	0.321	0.432	0.108	**0.554**	**0.032**
Mix TG (O-54:6)	**0.711**	**0.003**	**0.564**	**0.028**	**0.546**	**0.035**	0.507	0.054	**0.632**	**0.011**	**0.561**	**0.030**	**0.657**	**0.008**	0.418	0.121	**0.711**	**0.003**	**0.589**	**0.021**	0.407	0.132	**0.514**	**0.050**	0.489	0.064	**0.696**	**0.004**
Mix TG (38:0)	**0.514**	**0.050**	0.507	0.054	**0.539**	**0.038**	**0.532**	**0.041**	**0.693**	**0.004**	0.393	0.147	**0.600**	**0.018**	0.321	0.243	**0.568**	**0.027**	**0.564**	**0.028**	0.107	0.704	0.407	0.132	0.225	0.420	**0.639**	**0.010**
Mix TG (O-57:8); O	**0.782**	**0.001**	**0.693**	**0.004**	**0.679**	**0.005**	**0.596**	**0.019**	**0.621**	**0.013**	**0.607**	**0.016**	**0.682**	**0.005**	0.432	0.108	**0.757**	**0.001**	**0.564**	**0.028**	0.404	0.136	0.496	0.060	**0.521**	**0.046**	**0.593**	**0.020**
Mix TG (O-53:7)	**0.564**	**0.028**	**0.521**	**0.046**	**0.539**	**0.038**	0.457	0.087	**0.593**	**0.020**	0.350	0.201	0.507	0.054	0.250	0.369	**0.514**	**0.050**	0.421	0.118	0.171	0.541	0.321	0.243	0.211	0.451	**0.575**	**0.025**
Mix TG (O-55:8)	**0.636**	**0.011**	**0.607**	**0.016**	**0.614**	**0.015**	**0.561**	**0.030**	**0.732**	**0.002**	**0.550**	**0.034**	**0.546**	**0.035**	**0.525**	**0.044**	**0.539**	**0.038**	**0.568**	**0.027**	0.411	0.128	0.482	0.069	0.389	0.152	**0.611**	**0.016**
Mix TG (60:9)	-0.375	0.168	-0.443	0.098	-0.468	0.079	-0.489	0.064	**-0.561**	**0.030**	-0.343	0.211	-0.461	0.084	-0.261	0.348	-0.443	0.098	-0.461	0.084	-0.029	0.919	-0.264	0.341	-0.200	0.475	-0.375	0.168
Mix TG (O-55:11); O	-0.393	0.147	-0.507	0.054	-0.475	0.074	**-0.589**	**0.021**	**-0.546**	**0.035**	-0.371	0.173	-0.486	0.066	-0.286	0.302	-0.425	0.114	-0.461	0.084	0.004	0.990	-0.357	0.191	-0.229	0.413	-0.346	0.206
Mix TG (O-59:11); O	-0.339	0.216	-0.475	0.074	-0.486	0.066	**-0.514**	**0.050**	-0.457	0.087	-0.332	0.226	-0.443	0.098	-0.214	0.443	-0.429	0.111	-0.382	0.160	0.007	0.980	-0.214	0.443	-0.200	0.475	-0.257	0.355
Mix TG (54:4)	-0.482	0.069	**-0.614**	**0.015**	**-0.611**	**0.016**	**-0.632**	**0.011**	**-0.532**	**0.041**	-0.418	0.121	**-0.546**	**0.035**	-0.257	0.355	**-0.536**	**0.040**	-0.436	0.104	-0.046	0.869	-0.293	0.289	-0.279	0.315	-0.321	0.243
Mix TG (58:6)	-0.339	0.216	-0.475	0.074	-0.486	0.066	**-0.514**	**0.050**	-0.457	0.087	-0.332	0.226	-0.443	0.098	-0.214	0.443	-0.429	0.111	-0.382	0.160	0.007	0.980	-0.214	0.443	-0.200	0.475	-0.257	0.355
Mix TG (55:3); O	-0.339	0.216	-0.429	0.111	-0.443	0.098	-0.504	0.056	**-0.532**	**0.041**	-0.318	0.248	-0.450	0.092	-0.218	0.435	-0.407	0.132	-0.461	0.084	0.021	0.940	-0.257	0.355	-0.200	0.475	-0.329	0.232
Mix TG (54:5)	**-0.586**	**0.022**	**-0.693**	**0.004**	**-0.689**	**0.004**	**-0.725**	**0.002**	**-0.632**	**0.011**	**-0.518**	**0.048**	**-0.639**	**0.010**	-0.357	0.191	**-0.618**	**0.014**	**-0.532**	**0.041**	-0.132	0.639	-0.393	0.147	-0.379	0.164	-0.429	0.111
TG(O-37:9); O2	**-0.614**	**0.015**	**-0.636**	**0.011**	**-0.646**	**0.009**	**-0.679**	**0.005**	**-0.782**	**0.001**	**-0.689**	**0.004**	**-0.686**	**0.005**	**-0.671**	**0.006**	**-0.707**	**0.003**	**-0.779**	**0.001**	-0.439	0.101	**-0.639**	**0.010**	**-0.564**	**0.028**	**-0.593**	**0.020**
TG(O-39:9); O2	**-0.614**	**0.015**	**-0.732**	**0.002**	**-0.729**	**0.002**	**-0.761**	**0.001**	**-0.664**	**0.007**	**-0.696**	**0.004**	**-0.675**	**0.006**	**-0.621**	**0.013**	**-0.679**	**0.005**	**-0.654**	**0.008**	-0.439	0.101	**-0.586**	**0.022**	**-0.586**	**0.022**	-0.432	0.108
Diacylglycerols																												
Mix DG (33:1)	**0.593**	**0.020**	0.496	0.060	**0.521**	**0.046**	0.457	0.087	**0.693**	**0.004**	0.450	0.092	**0.596**	**0.019**	0.375	0.168	**0.604**	**0.017**	**0.568**	**0.027**	0.282	0.308	0.421	0.118	0.293	0.289	**0.704**	**0.003**
Mix DG (35:1)	**0.536**	**0.040**	0.425	0.114	0.461	0.084	0.379	0.164	**0.618**	**0.014**	0.414	0.125	**0.539**	**0.038**	0.329	0.232	**0.554**	**0.032**	0.496	0.060	0.293	0.289	0.350	0.201	0.279	0.315	**0.693**	**0.004**
DG (37:2)	0.496	0.060	0.350	0.201	0.379	0.164	0.311	0.260	**0.539**	**0.038**	0.432	0.108	0.475	0.074	0.386	0.156	0.486	0.066	0.439	0.101	0.421	0.118	0.386	0.156	0.332	0.226	**0.668**	**0.007**
DG (36:3)	-0.500	0.058	**-0.557**	**0.031**	**-0.564**	**0.028**	**-0.643**	**0.010**	**-0.579**	**0.024**	-0.457	0.087	**-0.579**	**0.024**	-0.364	0.182	**-0.536**	**0.040**	**-0.557**	**0.031**	-0.139	0.621	-0.393	0.147	-0.379	0.164	-0.282	0.308
DG (36:4)	**-0.536**	**0.040**	**-0.564**	**0.028**	**-0.579**	**0.024**	**-0.632**	**0.011**	**-0.682**	**0.005**	-0.496	0.060	**-0.579**	**0.024**	-0.464	0.081	**-0.539**	**0.038**	**-0.629**	**0.012**	-0.207	0.459	-0.496	0.060	-0.379	0.164	-0.396	0.143
Sphingomyelins																												
SM (40:1);O2	**0.746**	**0.001**	**0.675**	**0.006**	**0.679**	**0.005**	**0.714**	**0.003**	**0.764**	**0.001**	**0.550**	**0.034**	**0.750**	**0.001**	0.436	0.104	**0.700**	**0.004**	**0.679**	**0.005**	0.246	0.376	**0.564**	**0.028**	0.439	0.101	**0.625**	**0.013**
Phosphatidylcholines																												
PC (40:5)	**0.886**	**<0.001**	**0.768**	**0.001**	**0.764**	**0.001**	**0.732**	**0.002**	**0.732**	**0.002**	**0.721**	**0.002**	**0.800**	**<0.001**	**0.575**	**0.025**	**0.800**	**<0.001**	**0.671**	**0.006**	0.511	0.052	**0.668**	**0.007**	**0.629**	**0.012**	**0.718**	**0.003**
PC (40:7)	-0.354	0.196	-0.421	0.118	-0.457	0.087	-0.475	0.074	**-0.532**	**0.041**	-0.486	0.066	-0.404	0.136	-0.439	0.101	-0.464	0.081	**-0.575**	**0.025**	-0.261	0.348	-0.339	0.216	-0.468	0.079	-0.254	0.362
PC (42:7)	**-0.621**	**0.013**	**-0.657**	**0.008**	**-0.682**	**0.005**	**-0.707**	**0.003**	**-0.786**	**0.001**	**-0.632**	**0.011**	**-0.682**	**0.005**	**-0.561**	**0.030**	**-0.686**	**0.005**	**-0.718**	**0.003**	-0.343	0.211	**-0.518**	**0.048**	-0.511	0.052	**-0.575**	**0.025**
Dihydro-Phosphatidylcholines																												
DihydroPC (35:0); O5	**0.800**	**<0.001**	**0.704**	**0.003**	**0.679**	**0.005**	**0.639**	**0.010**	**0.561**	**0.030**	**0.607**	**0.016**	**0.704**	**0.003**	0.450	0.092	**0.700**	**0.004**	0.475	0.074	0.457	0.087	**0.564**	**0.028**	**0.514**	**0.050**	**0.632**	**0.011**
Carnitines																												
CAR (15:0)	**0.811**	**<0.001**	**0.732**	**0.002**	**0.682**	**0.005**	**0.771**	**0.001**	**0.611**	**0.016**	**0.604**	**0.017**	**0.832**	**<0.001**	0.336	0.221	**0.786**	**0.001**	**0.550**	**0.034**	0.264	0.341	0.454	0.089	**0.536**	**0.040**	**0.607**	**0.016**
CAR (14:1); O	**-0.629**	**0.012**	**-0.682**	**0.005**	**-0.700**	**0.004**	**-0.721**	**0.002**	**-0.725**	**0.002**	**-0.693**	**0.004**	**-0.639**	**0.010**	**-0.632**	**0.011**	**-0.661**	**0.007**	**-0.718**	**0.003**	-0.425	0.114	**-0.571**	**0.026**	**-0.607**	**0.016**	-0.414	0.125
CAR (18:2); O	**-0.543**	**0.037**	**-0.600**	**0.018**	**-0.621**	**0.013**	**-0.593**	**0.020**	**-0.675**	**0.006**	**-0.525**	**0.044**	**-0.536**	**0.040**	-0.468	0.079	**-0.557**	**0.031**	**-0.586**	**0.022**	-0.243	0.383	-0.450	0.092	-0.379	0.164	-0.436	0.104
CAR (16:1); O	-0.468	0.079	**-0.546**	**0.035**	**-0.543**	**0.037**	**-0.575**	**0.025**	**-0.611**	**0.016**	-0.475	0.074	-0.489	0.064	-0.436	0.104	-0.489	0.064	**-0.557**	**0.031**	-0.179	0.524	-0.446	0.095	-0.339	0.216	-0.311	0.260
CAR (18:1); O	-0.500	0.058		**0.040**	**-0.543**	**0.037**	**-0.557**	**0.031**	**-0.596**	**0.019**	-0.464	0.081	-0.482	0.069	-0.421	0.118	-0.493	0.062	**-0.554**	**0.032**	-0.171	0.541	-0.439	0.101	-0.357	0.191	-0.300	0.277
